# The Impact of Physical Activity on Clinical Outcomes in Children with Cystic Fibrosis: A Narrative Review

**DOI:** 10.3390/children12070831

**Published:** 2025-06-23

**Authors:** Chiara Rosolia Capasso, Antonio Luca Miniato, Paola Di Filippo, Armando Di Ludovico, Sabrina Di Pillo, Francesco Chiarelli, Giuseppe Francesco Sferrazza Papa, Marina Attanasi

**Affiliations:** 1 Pediatric Allergy and Pulmonology Unit, Department of Pediatrics, University of Chieti-Pescara, Via dei Vestini n°5, 66100 Chieti, Italy; chiara.rosoliacapasso@studenti.unich.it (C.R.C.); antonioluca.miniato@studenti.unich.it (A.L.M.); paola.difilippo@asl2abruzzo.it (P.D.F.); armando.diludovico@studenti.unich.it (A.D.L.); sabrina.dipillo@asl2abruzzo.it (S.D.P.); chiarelli@unich.it (F.C.); 2 Department of Neurorehabilitation Sciences, Casa di Cura Igea, 20144 Milan, Italy; g.sferrazza@casacuraigea.it

**Keywords:** training, exercise, aerobic fitness, pulmonary function, muscle strength, physical fitness, adolescents

## Abstract

Background: Cystic fibrosis (CF) is a chronic genetic disease marked by progressive lung function decline and increased respiratory infections. Emerging evidence supports the role of physical exercise in improving lung function, aerobic capacity, and quality of life in pediatric CF patients. Methods: We reviewed randomized clinical trials and observational studies from the last ten years, sourced from PubMed and Google Scholar. Included studies involved children and adolescents (0–18 years) with CF and assessed physical exercise as a primary intervention to improve lung function, aerobic fitness, quality of life, or hospitalization rates. Results: Aerobic training, particularly when combined with strength training, improves cardiorespiratory fitness and muscle strength without compromising nutritional status. High-Intensity Interval Training and Inspiratory Muscle Training show potential but need further validation. Supervised, personalized exercise programs are key to promoting adherence and optimizing outcomes. Conclusions: Exercise-based interventions in pediatric CF should evolve toward personalized, technology-enhanced, and sustainable models. Integrating wearable devices, adapting programs to individual needs, and leveraging early parental involvement may enhance engagement and outcomes, especially in the era of CFTR modulator therapies.

## 1. Introduction

Cystic Fibrosis (CF) is an autosomal recessive hereditary disorder resulting from mutations in the CF Transmembrane Conductance Regulator (CFTR) gene, located on chromosome 7 [1]. These mutations lead to a defect in the chloride channel, affecting all epithelial cells, disrupting ion transport across various tissues, and causing blockage of secretory glands [2]. Although CF affects multiple organs [3], pulmonary involvement is the primary factor influencing both disease severity and patient survival [4].

First identified in 1938 by pathologist Dorothy Hansine Andersen, CF was initially a fatal condition in early childhood [5,6]. However, due to advancements in medical treatments, patient life expectancy has significantly improved, making childhood mortality an increasingly rare occurrence [6,7,8]. Today, CFTR modulator therapies are revolutionizing the prognosis for people with CF. Nonetheless, lifelong dependence on a combination of these drugs is not an ideal solution due to high costs and potential long-term treatment intolerance in some patients. Consequently, alternative approaches, such as physical exercise, are being actively investigated [9]. Regular physical activity appears to offer numerous potential benefits in the management of CF, including improvements in pulmonary function [10], enhanced mucus clearance [11], improved bone health [12] optimized body composition [13], reduced systemic inflammation [14], and increased overall physical fitness [15]. As a result, exercise is now recognized as an effective non-pharmacological strategy to achieve optimal physical condition, ensuring that patients can undergo new therapeutic treatments under the best possible health conditions [16].

This review offers an updated overview of the impact of physical exercise on children with CF, offering guidance to inform future therapeutic strategies and clinical guidelines.

While several systematic reviews and a recent Cochrane review have evaluated the role of exercise in people with CF, these often include mixed adult and children, and do not fully reflect recent changes in CF management—such as the widespread use of CFTR modulators and the increasing role of digital and personalized approaches. This narrative review aims to provide an updated and focused synthesis of recent interventional studies on physical activity in children and adolescents with CF, highlighting the most effective training modalities and outlining future directions in personalized, sustainable, and technology-integrated interventions.

## 2. Materials and Methods

We conducted a narrative review of original interventional studies examining the effects of physical exercise on clinical outcomes in children and adolescents with cystic fibrosis (CF). A systematic search was performed using PubMed and Google Scholar for articles published between January 2010 and March 2025. Search terms included combinations of the following keywords: cystic fibrosis, training, exercise, aerobic fitness, pulmonary function, muscle strength, physical fitness, children, adolescents. 

Studies were included if they met the following criteria: (1) peer-reviewed original articles written in English; (2) study population aged 0–18 years with a diagnosis of CF; (3) exercise-based interventions (aerobic, anaerobic, or combined) as the primary treatment strategy; and (4) reported outcomes related to pulmonary function, aerobic capacity, muscle strength, nutritional status, or hospitalization frequency. 

Exclusion criteria included: (1) studies involving exclusively adults or without stratified pediatric data; (2) studies focused solely on respiratory physiotherapy (e.g., airway clearance techniques) without an exercise component; (3) reviews, meta-analyses, or duplicate publications; and (4) case reports, abstracts, or opinion papers. 

**Figure 1:**
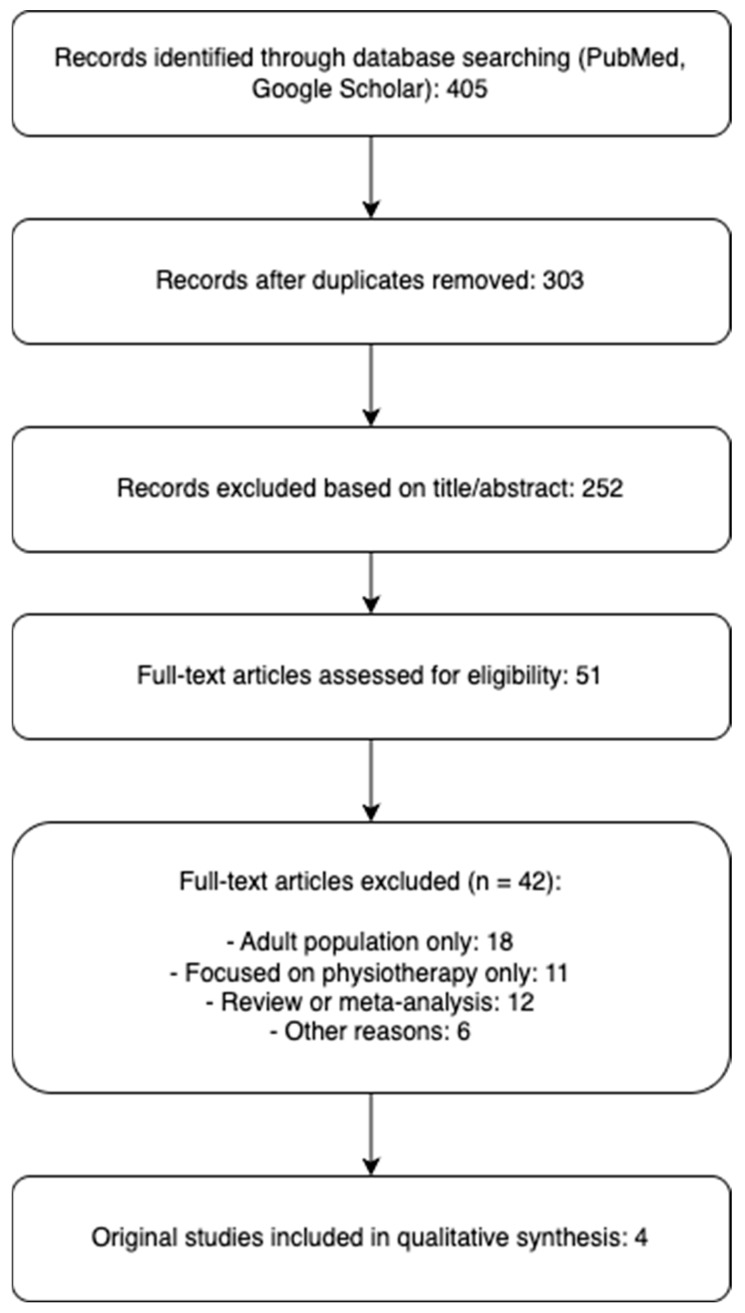
Flow-chart of the studies included.

After removing duplicates, 303 records were screened by title and abstract. 51 full-text articles were assessed, of which 42 were excluded for not meeting the inclusion criteria. Ultimately, 4 original studies were included in the final analysis. The updated study selection process is illustrated in the revised PRISMA flow diagram. The flow-chart of the studies included is showed in Figure 1.

## 3. Results

The studies included are summarized in Table 1. 

Zeren et al. [17] conducted a randomized controlled trial in 2019 involving a population of 26 children aged 8 to 18 years with CF, aiming to assess whether the addition of Inspiratory Muscle Training (IMT) to standard chest physiotherapy would improve outcomes in these patients. Although both groups showed improvements in pulmonary function, postural stability, and functional capacity, only the group that received IMT demonstrated a significantly greater increase in inspiratory muscle strength, suggesting that IMT may be particularly beneficial for patients with respiratory muscle weakness. 

Elbasan et al. [18] conducted a clinical trial involving a population of 16 patients with CF between the ages 5–14 years. This study evaluated a 6-week program combining chest physiotherapy and aerobic exercise, resulting in significant improvements in thoracic mobility, muscle endurance, strength, and cardiovascular fitness. Despite the small sample size and absence of a control group, the findings support early inclusion of aerobic training in CF management to enhance physical fitness and respiratory function.

Gruber et al. [19] conducted a clinical trial evaluating a 12-month exercise program in six children with CF, aged 6 to 14 years (mean age 11.3 years), all with normal lung function (mean FEV_1_ 102.5% predicted). During the first 6 months of monitoring, slight improvements in aerobic capacity were observed, which partially declined after the monitoring ended. The results suggest that regular monitoring and close contact with exercise specialists are essential to maintain motivation and promote lasting benefits in the physical fitness of children with CF. 

Mackintosh et al. [20] conducted a clinical trial involving a population of 18 children with mild to moderate CF (10 boys; mean age 12.4 ± 2.8 years) and 18 healthy controls (10 boys; mean age 12.5 ± 2.7 years). This study investigated physical activity levels (PAL) and patterns in children with CF compared to healthy age- and sex-matched controls. The conclusion is that despite the lack of differences between CF and healthy children, there is still a need for strategies to increase physical activity in the CF population given the numerous additional health benefits beyond those observed in healthy children.

Other systematic reviews and meta-analyses on this topic, including the Cochrane review, are discussed in the Discussion section to provide context and comparison with the original studies analyzed here.

## 4. Discussion

### 4.1. Aerobic Exercise

One of the most important themes emerging from this review is the role of aerobic exercise. Aerobic exercise plays a key role in the management of CF, particularly in improving cardiorespiratory fitness. Many studies have investigated the effects of aerobic activity in children and adolescents with CF, showing encouraging results, albeit with some limitations.

Elbasan et al. [18] demonstrated that a program combining active breathing techniques with aerobic treadmill exercise, performed three times a week for six weeks, led to significant improvements in thoracic mobility, muscular endurance and cardiovascular performance in children with CF. These findings highlight the importance of early integration of aerobic activity in the treatment of these patients.

Similarly, Gruber et al. [19] observed slight improvements in aerobic capacity (VO_2_ peak and peak workload) following a six-month monitored exercise program, although these improvements were not statistically significant. Interestingly, the benefits were partly maintained even after the monitoring period ended, suggesting a lasting effect of aerobic exercise, especially when supported by regular and personalized monitoring.

### 4.2. Combination of Aerobic and Strength/Anaerobic Training

According to the findings of our review, the combined approach integrating aerobic and anaerobic (resistance/strength) exercises proves to be particularly effective in young individuals with CF, yielding broader benefits compared to adopting only one type of exercise.

Williams et al. [21] highlighted that aerobic training promotes oxygen utilization and mucus clearance, while anaerobic training increases muscle mass and strength. Therefore, combining these two exercise modalities offers superior advantages, both physiologically and in terms of the patient’s quality of life.

This concept was further confirmed by the more recent review by Thorel et al. [22], which reported significant improvements in muscle strength in both lower and upper limbs following programs that integrated aerobic and strength training. In addition to strength gains, increases in muscle mass and maximal oxygen consumption were also observed—key indicators of enhanced overall physical capacity.

In conclusion, the combination of aerobic and strength exercises represents a highly effective strategy for improving both muscular fitness and cardiorespiratory capacity in young people with CF.

### 4.3. High- Intensity Interval Training

The role of HIIT in patients with CF is still under exploration, but early studies suggest that it may be a promising training modality. Garcia-Perez-de-Sevilla et al. [23] reported that HIIT-based programs, as well as strength training, are potentially the most effective in improving fitness in CF patients, although few studies have directly evaluated these approaches.

HIIT, characterized by short bursts of high-intensity exercise alternated with recovery periods, may be better tolerated than continuous training, especially in patients with respiratory limitations. According to the authors, HIIT may promote superior physiological adaptations compared to traditional exercise, thus representing a valuable alternative worthy of further investigation.

### 4.4. Inspiratory Muscle Training

IMT specifically aims to strengthen the inspiratory muscles, mostly the diaphragm, and is sometimes proposed as a supplement to traditional respiratory physiotherapy in patients with CF.

Among field studies, the work by Zeren et al. [17] was particularly noteworthy. They conducted a randomized trial on children with CF, comparing outcomes between those who underwent only respiratory physiotherapy and those who combined it with IMT. The results showed that IMT led to a significant improvement in inspiratory muscle strength (measured as maximal inspiratory pressure, MIP), with an increase of 38 cmH_2_O compared to 13 cmH_2_O in the group without IMT. However, no additional benefits were observed in terms of pulmonary function, postural stability, or functional capacity (as measured by the 6-min walk test) compared to physiotherapy alone.

Therefore, IMT may be beneficial for patients with significant respiratory muscle weakness, but it does not appear to offer generalized additional benefits in children with CF who already have normal pulmonary function.

### 4.5. Close Contact and Personalized Approaches

Close contact and personalized approaches are crucial for effective and sustainable exercise interventions in individuals with CF, especially in children and adolescents.

Gruber et al. [19] found that monitoring seems to facilitate the achievement of beneficial effects on physical fitness in children with CF. They recommend implementing ongoing personalized exercise monitoring programs with regular support from an exercise therapist to sustain long-term motivation and engagement. The biweekly telephone contact in their study served to address problems, motivate participants, and highlight successes, indicating the importance of this close support.

The study by Gruber et al. [19] also highlights that home-based exercise programs with regular contact with an exercise therapist yield larger effects compared to those with less contact. They assume that close contact between exercise scientists or physiotherapists is an important factor for motivating individuals with CF to participate and maintain regular physical activity, particularly for children. Their results showed a positive influence between close supervision by experienced sports therapists and improvements in VO_2_ and habitual physical activity in children with CF.

Radtke et al. [24] in their Cochrane review note that the benefits of physical activity may be influenced by individual preferences and barriers. They emphasize the need for healthcare professionals to provide advice and guidance addressing individual barriers and facilitators to long-term participation. Furthermore, they suggest that participation rates and adherence, which are often suboptimal, could be improved by considering individual facilitators and barriers to build positive, long-term physical activity behavior.

Williams et al. [21] recommend that exercise training should be adapted to the special needs and preferences of each individual. They also point out that detailed exercise testing is recommended prior to training to provide safe and personalized recommendations. Thorel et al. [22] suggest that while supervised exercise is intuitively suitable for those with muscle dysfunction, a major issue for others is finding ways to engage in long-term physical activity, implying the need for tailored approaches beyond just structured training.

Finally, Gruber et al. [19] and Williams et al. [21] underscore the importance of early education for parents about the positive effects of physical activity and the need for their support and encouragement. This collaborative approach strengthens the impact of personalized interventions.

### 4.6. Forced Expiratory Volume in One Second

The effect of regular exercise on FEV_1_ in individuals with CF is questionable, as the evidence suggests that significant improvements in FEV_1_ are unlikely to occur solely due to exercise. 

A Cochrane review by Radtke et al. [24] found low-certainty evidence that a physical activity intervention compared to a control group has little or no effect on FEV_1_% predicted. Their analysis of multiple studies with active interventions up to six months did not show a significant difference in FEV_1_ between the groups. Similarly, analyses of follow-up periods in the Cochrane review did not reveal significant differences in FEV_1_. The overall conclusion of the Cochrane review was that while physical activity and exercise training likely lead to a slightly greater improvement in exercise capacity, they might have little or no effect on pulmonary function as measured by FEV_1_.

The review by García-Pérez-de-Sevilla et al. [12] notes that few studies have analyzed both lung function and physical fitness as target variables. Their review includes several studies where FEV_1_ remained unchanged in the intervention group after exercise programs in both children and adults. They specifically mention that one study in adults with CF observed no improvement in FEV_1_.

Thorel et al. [22] study shows that many studies reported no significant difference in FEV_1_ between the exercise intervention and control groups.

Gruber et al. [19] mention that improvements in exercise capacity have been observed without corresponding increases in ppFEV_1_, suggesting that the benefits of exercise in CF may primarily lie in areas other than directly improving FEV_1_.

In conclusion, while exercise is widely recommended and beneficial for individuals with CF for improving physical fitness, muscle strength, and overall well-being, the current evidence from these sources indicates that regular exercise does not typically lead to a significant or consistent increase in FEV_1_. The Cochrane review provides the strongest evidence suggesting little to no effect on FEV_1_% predicted.

### 4.7. Nutritional Status

A key theme of this review is the Nutritional status, as it is a primary focus in the care of individuals with CF. It is commonly assessed using anthropometric measures, mainly body mass index (BMI) for adults or BMI percentile for children. BMI has been identified as independent predictor of mortality in CF, and a low BMI is associated with decreased pulmonary function (FEV_1_). Current CF guidelines recommend specific BMI targets for children and adults.

A systematic review by Nicolson et al. [25] aimed to investigate the effect of exercise on measures of nutritional status in children and adults with CF. Their review included four eligible studies, all of which involved only children. These studies indicated that exercise training did not worsen nutritional status. In fact, two studies that included resistance exercise reported an increase in fat-free mass. While changes in BMI and BMI z-scores were not clinically or statistically significant, body mass increased over time in both the intervention and control groups in the majority of studies. Notably, the resistance exercise training (RET) group in one study [26] experienced statistically significant and clinically meaningful increases in body mass (7.25%), entirely due to an increase in fat-free (muscle) mass. Although often statistically insignificant, fat-free mass tended to increase in the exercise groups and decrease in the control groups.

The review by Nicolson et al. [25] concluded that there is no evidence that aerobic exercise training (AET) or RET will worsen an individual’s nutritional status, even in normal to underweight patients. RET may actually support the maintenance or enhancement of body weight, particularly lean mass. Healthcare providers should reassure patients who are worried about the possible negative effects of exercise on their nutritional status and body composition that physical activity is not harmful—in fact, it may have beneficial effects. It is essential for the CF care team to collaborate with registered dietitians to develop tailored nutrition plans that align with patients’ exercise routines and support their individual goals related to weight and body composition. Additionally, both AET and RET provide further advantages for individuals with CF, including improvements in aerobic fitness and muscular strength, which are linked to better health outcomes.

**Figure 2:**
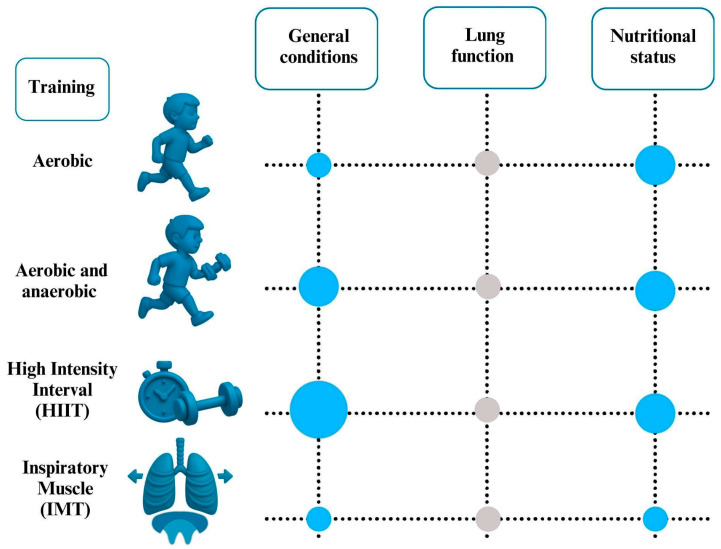
The main findings of the studies reviewed. Positive (blue) and irrelevant (grey) relationship between type of training and outcomes in children and adolescents with CF.

All the key findings of this review are presented in Figure 2.

This narrative review synthesized evidence from four original studies evaluating the impact of physical activity interventions in pediatric populations with cystic fibrosis (CF). The included studies focused on various forms of exercise, including aerobic training, inspiratory muscle training, and structured monitoring of free-living activity. Despite the small number of studies and the heterogeneity of designs, consistent benefits were reported in terms of physical fitness, muscle strength, and aerobic capacity.

To contextualize our findings, it is important to reference broader systematic reviews and meta-analyses, including the Cochrane review by Radtke et al. [24], which examined 24 RCTs involving both adults and children with CF. Their findings indicated that exercise interventions likely improve exercise capacity—particularly when sustained for over six months—but have limited or no effect on lung function (FEV_1_) or health-related quality of life. Similarly, Thorel et al. [22] and Garcia-Perez-de-Sevilla et al. [23] emphasized the benefits of combined strength and aerobic training on peripheral muscle strength, while also highlighting the methodological limitations and heterogeneity in the literature. Our synthesis aligns with these conclusions, reinforcing that while direct improvements in lung function are limited, structured exercise programs tailored to pediatric CF populations can improve physical performance and engagement. These benefits are especially relevant in the context of early intervention and the growing role of digital support strategies. Elbasan et al. [18] demonstrated that a program combining active breathing techniques with aerobic treadmill exercise, performed three times a week for six weeks, led to significant improvements in thoracic mobility, muscular endurance and cardiovascular performance in children with CF. These findings highlight the importance of early integration of aerobic activity in the treatment of these patients. Similarly, Gruber et al. [19] observed slight improvements in aerobic capacity (VO_2_ peak and peak workload) following a six-month monitored exercise program, although these improvements were not statistically significant. Interestingly, the benefits were partly maintained even after the monitoring period ended, suggesting a lasting effect of aerobic exercise, especially when supported by regular and personalized monitoring.

## 5. Limitations

The most important limitation of this review is the small number of studies included (N = 4 articles). Additionally, the selected studies exhibit several intrinsic limitations. Specifically, the clinical trial by Elbasan et al. [18] faces limitations such as a small sample size (only 16 children with CF), the absence of a control group for comparison, the potential for a learning effect in the physical fitness tests used, and no long-term follow-up to assess whether improvements were sustained. Furthermore, the study included only clinically stable CF patients, which limits the generalizability of the results to those with more severe disease.

The clinical trial by Gruber et al. [19] also presents notable limitations, including a very small sample size (only six children completed the full program), no control group, selection bias (as participants had normal lung function and were clinically stable), difficulty obtaining accurate feedback on physical activities due to incomplete exercise diaries, and varied adherence to the recommended exercise program.

The meta-analysis by Garcia-Perez-de-Sevilla et al. [23] is limited by a small number of RCTs analyzing the same variables with consistent measurements, as well as small sample sizes in most of the studies. Furthermore, few studies analyzed pulmonary, cardiorespiratory, and muscular functions simultaneously, and the heterogeneity in the types and doses of exercise interventions complicates the interpretation of results. The inclusion of unsupervised programs in some studies also presents potential risks of bias.

In the systematic review by Nicolson et al. [25] the authors highlighted the lack of high-quality research on this topic, noting that future research should focus on longer-term outcomes and examine the effects on underweight, normal weight, and overweight CF patients. Similarly, Thorel et al. [22] emphasized the very low quality of the evidence according to the GRADE system, with poor certainty in the findings. They called for high-quality randomized controlled studies to validate the results and underscored the need for standardized muscle strength measurement protocols in CF patients. Furthermore, they suggested that future research should focus on patients with established muscle dysfunction or reduced endurance, use standardized protocols, and explore strategies to promote long-term engagement in physical activity.

Moreover the small number of included studies, their clinical and methodological heterogeneity (in terms of intervention type, outcome measures, and duration), and the lack of comparable endpoints (e.g., some report VO_2_ peak, others FEV_1_, others qualitative adherence outcomes) currently limit the feasibility of a meaningful quantitative synthesis.

## 6. Conclusions and Future Perspectives

The future of physical exercise interventions for children with CF appears promising and is likely to be shaped by several key trends and considerations.

- Personalized and Tailored Interventions: future directions emphasize the need for more personalized exercise programs [19,21]. This includes considering individual fitness levels, disease severity, preferences, and barriers to participation [24]. Future research should focus on identifying which exercise programs are most effective for specific subgroups of children with CF [23,24].
- Integration of Technology: The use of wearable technology like fitness trackers and step counters, combined with motivational feedback and goal setting, is seen as a promising approach for future physical activity interventions [19,24]. Technology can help monitor activity levels, improve adherence, and provide personalized feedback.
- Impact of CFTR Modulator Therapies: The advent of highly effective CFTR modulator therapies will likely have a significant impact on how exercise is approached [22,24]. As the nutritional, respiratory, and physical status of children with CF is better preserved due to these therapies, the focus of exercise interventions might evolve to optimize overall fitness and long-term health, rather than solely compensating for disease-related limitations [22]. Research is needed to understand how these therapies influence daily physical activity and exercise behavior [24].
- Focus on Long-Term Adherence and Sustainability: Future efforts will need to prioritize strategies that promote long-term engagement in physical activity and make exercise a part of the patient’s lifestyle [19,22,23]. This may involve incorporating elements of behavior change theory and addressing factors like motivation, self-efficacy, and perceived enjoyment [22,24]. Interventions that are fun and adaptable to daily life, such as video game-based training, might play a larger role [22].
- Combining Different Training Modalities: Future studies should explore the benefits of combining strength exercise with HIIT as these have shown promising results [23] Further research is needed to determine the optimal combination and dosage of aerobic and anaerobic training for children with CF [21].
- Early Intervention and Parental Involvement: Encouraging an active lifestyle early in life and involving parents as role models will be crucial for establishing healthy habits [19,21] Educating parents about the benefits of physical activity and providing them with support will be essential.
- Addressing Muscle Function: Given that exercise training can improve peripheral muscle strength in young individuals with CF [22], future research should further investigate the most effective strategies to enhance muscle function and address potential muscle dysfunction from an early age.
- Further High-Quality Research: There is a continued need for high-quality, sufficiently sized randomized controlled trials with longer follow-up periods to comprehensively assess the benefits of different exercise interventions in children with CF [21,22,23,24]. These studies should analyze a wider range of outcomes, including lung function, exercise capacity, muscle strength, nutritional status, and quality of life. Research should also investigate the dose-response relationships between physical activity and clinical outcomes [24].
- The integration of eHealth technologies, such as wearable fitness trackers and app-based feedback systems, shows promising potential in pediatric CF care. Such tools have already proven effective in pediatric asthma management by enhancing motivation, adherence, and health outcomes, as reported by van der Kamp et al. [26]. In CF, these technologies could facilitate the transition toward more autonomous, personalized activity plans. However, as shown by Berghea et al. [27], the adoption of AI-based digital tools may be influenced by parental educational level and access to resources—factors that must be addressed to ensure equitable implementation. Nonetheless, parents of children with chronic diseases like CF may show greater openness to these tools compared to other pediatric populations, representing an opportunity for early integration.

In summary, the future of exercise for children with CF will likely involve highly personalized interventions leveraging technology, integrated with new therapeutic advancements, and focused on fostering long-term active lifestyles through engaging and sustainable strategies; all underpinned by rigorous scientific research.

## Figures and Tables

**Table 1:** The main characteristics of the included original studies in the review.

Outcomes and Conclusions	Objective	Type of Training	Study Population	Study Design	Year	Authors
IMT group showed greater increase in inspiratory muscle strength. IMT may benefit those with respiratory muscle weakness.	Assess IMT addition to standard physiotherapy.	Chest physiotherapy ± Inspiratory Muscle Training	N = 36, aged 8–18 years ys	RCT	2019	Zeren et al. [17]
Improved thoracic mobility, muscle endurance, and cardiovascular performance.	Evaluate impact on thoracic mobility and physical fitness.	Aerobic exercise + chest physiotherapy	N = 16, aged 5–14 years	CT	2012	Elbasan et al. [18]
Slight aerobic capacity improvements; maintained post-monitoring. Importance of therapist contact for adherence.	Investigate aerobic training effects over 12 months.	Monitored aerobic exercise program	N = 6, aged 6–14 years (mean FEV_1_ 102%)	One-group pre-post intervention study	2022	Gruber et al. [19]
Similar activity patterns; underscores need for promoting physical activity among CF patients.	Compare activity levels between CF patients and healthy peers.	Free-living activity pattern analysis	N = 18 CF children and N = 18 controls, mean age ~12 years	CT	2018	Mackintosh et al. [20]

CF: cystic fibrosis, RCT: randomized controlled trials; CT: clinical trial; IMT: Inspiratory Muscle Training.

## Data Availability

Not applicable.

## Abbreviations

Abbreviations used in this manuscript:

CF	Cystic Fibrosis
HIIT	High-Intensity Interval Training
IMT	Inspiratory Muscle Training
FEV_1_	Forced Expiratory Volume in 1 s
RCTs	randomized controlled trials
BMI	body mass index
RET	resistance exercise training
FVC	forced vital capacity
PEF	peak expiratory flow
CPETs	Cardiopulmonary Exercise Tests
PAL	physical activity levels
VO_2_	peak refers to the highest rate of oxygen consumption (VO_2_)
Wpeak	peak workload
HRQoL	Health-Related Quality of Life
SMD	standardized mean difference
low-LPA	low light physical activity
